# Effects of C60 Fullerene on Thioacetamide-Induced Rat Liver Toxicity and Gut Microbiome Changes

**DOI:** 10.3390/antiox10060911

**Published:** 2021-06-04

**Authors:** Siniša Đurašević, Snežana Pejić, Ilijana Grigorov, Gorana Nikolić, Dragana Mitić-Ćulafić, Milan Dragićević, Jelena Đorđević, Nevena Todorović Vukotić, Neda Đorđević, Ana Todorović, Dunja Drakulić, Filip Veljković, Snežana B. Pajović, Zoran Todorović

**Affiliations:** 1 Faculty of Biology, University of Belgrade, Studentski Trg 16, 11000 Belgrade, Serbia; mdragana@bio.bg.ac.rs (D.M.-Ć.); jelenadj@bio.bg.ac.rs (J.Đ.); 2 “Vinča” Institute of Nuclear Sciences, National Institute of the Republic of Serbia, University of Belgrade, Mike Petrovića Alasa 12–14, 11000 Belgrade, Serbia; snezana@vin.bg.ac.rs (S.P.); nevenat@vin.bg.ac.rs (N.T.V.); neda@vin.bg.ac.rs (N.Đ.); anato@vin.bg.ac.rs (A.T.); drakulic@vin.bg.ac.rs (D.D.); filipveljkovic@vin.bg.ac.rs (F.V.); pajovic@vin.bg.ac.rs (S.B.P.); 3 Institute for Biological Research “Siniša Stanković”, National Institute of Republic of Serbia, University of Belgrade, Bulevar Despota Stefana 142, 11060 Belgrade, Serbia; iligri@ibiss.bg.ac.rs (I.G.); mdragicevic@ibiss.bg.ac.rs (M.D.); 4 Faculty of Medicine, University of Belgrade, Dr. Subotića 8, 11000 Belgrade, Serbia; gorana.nikolic03@gmail.com (G.N.); zoran.todorovic@med.bg.ac.rs (Z.T.); 5 University Medical Centre “Bežanijska kosa”, University of Belgrade, Dr. Žorža Matea bb, 11080 Belgrade, Serbia

**Keywords:** thioacetamide, C60 fullerene, liver, oxidative stress, inflammation, gut microbiome

## Abstract

Thioacetamide (TAA) is widely used to study liver toxicity accompanied by oxidative stress, inflammation, cell necrosis, fibrosis, cholestasis, and hepatocellular carcinoma. As an efficient free radical’s scavenger, C60 fullerene is considered a potential liver-protective agent in chemically-induced liver injury. In the present work, we examined the hepatoprotective effects of two C60 doses dissolved in virgin olive oil against TAA-induced hepatotoxicity in rats. We showed that TAA-induced increase in liver oxidative stress, judged by the changes in the activities of SOD, CAT, GPx, GR, GST, the content of GSH and 4-HNE, and expression of HO-1, MnSOD, and CuZnSOD, was more effectively ameliorated with a lower C60 dose. Improvement in liver antioxidative status caused by C60 was accompanied by a decrease in liver HMGB1 expression and an increase in nuclear Nrf2/NF-κB p65 ratio, suggesting a reduction in inflammation, necrosis and fibrosis. These results were in accordance with liver histology analysis, liver comet assay, and changes in serum levels of ALT, AST, and AP. The changes observed in gut microbiome support detrimental effects of TAA and hepatoprotective effects of low C60 dose. Less protective effects of a higher C60 dose could be a consequence of its enhanced aggregation and related pro-oxidant role.

## 1. Introduction

Liver toxicity is becoming a growing problem due to the increased use of chemicals and exposure to xenobiotics in the environment. Unique metabolism, anatomy, and close connection with the gastrointestinal tract make the liver susceptible to harmful effects of such agents. For this reason, the study of hepatotoxicity is the focus of researchers whose goal is to develop a new pharmaceutical approach for the prevention and treatment of chemically induced liver injury [1]. Related to this, nanotechnology offers some perspective therapeutic strategies. Fullerene C60, a structurally specific class of carbon nanoparticles, is of great interest for biomedical applications [2]. Due to its size (0.72 nm in diameter), C60 can penetrate through the plasma membrane and act intracellularly as an efficient free-radical scavenger due to a large amount of conjugated double carbon bonds which can easily take up electrons [3,4], which suggests its hepatoprotective role when used in a dose-dependent manner [5,6]. However, the cellular action of C60 fullerene depends on many factors, especially the solvents used. C60 is sparingly soluble in many organic solvents [7]. To avoid toxicity, the use of biocompatible solvents, such as vegetable oils [8], is mandatory for its application in living systems [9]. The C60 solubility depends on the saturation level of vegetable oils, being higher in oils with a higher degree of unsaturated fatty acids [9]. As a result, the level of the chemical interactions between C60 and the fatty acid chains is higher in oils with a lower level of saturation [10].

Thioacetamide (TAA) is widely used to study liver toxicity and fibrosis. The pathogenesis of TAA-induced liver injuries includes oxidative stress, inflammation, necrosis, steatosis, fibrosis, cholestasis, hepatocellular carcinoma, hepatitis, and fatty liver [11]. Thioacetamide-S-oxide, an TAA intermediate metabolite, binds to hepatic macromolecules causing changes in cell permeability and Ca^2+^ uptake, leading to cellular damage hepatic necrosis [12]. TAA can generate many reactive oxygen species (ROS), which can overwhelm the antioxidant defence mechanism in the liver and damage cellular macromolecules, such as lipids, proteins, and DNA [13].

Since increased oxidative stress is the cornerstone of pathological mechanisms underpinning TAA-induced liver injury [14], we hypothesized that C60 fullerene, due to its strong antioxidant capacity, could be useful in the prevention of TAA-related liver toxicity. Therefore, in this study, we attempted to evaluate the dose-dependent hepatoprotective role of C60 fullerene dissolved in virgin olive oil (VOO) against TAA-induced liver oxidative stress injury. We investigated the liver activity of superoxide dismutase (SOD), catalase (CAT), glutathione peroxidase (GPx), glutathione reductase (GR), and glutathione S-transferase (GST), as well as the level of total glutathione (GSH) and 4-hydroxynonenal (4-HNE) content [15], along with the expression levels of nuclear factor (erythroid-derived 2)-like 2 (Nrf2), and nuclear factor-kappa B p65 (NF-κB p65), the two key transcription factors controlling many aspects of cell homeostasis in response to oxidative and toxic insults. We also investigated the expression levels of haeme oxygenase-1 (HO-1), an enzyme that plays a vital role in the catabolism of haem [16] and the expression of which is regulated by the Nrf2 [17], together with the expression levels of manganese superoxide dismutase (MnSOD) and copper-zinc superoxide-dismutase (CuZnSOD), two antioxidant enzymes known to be regulated by NF-κB p65 and Nrf2 [18]. Since TAA is a well-known hepatotoxicant that produces centrilobular necrosis in experimental animals accompanied by a massive inflammatory reaction, we examined the expression level of the liver high mobility group box 1 (HMGB1) protein, a necrotic marker and an inflammatory mediator due to its ability to activate NF-κB p65 signalling. As additional markers of hepatic injury, we investigated the liver histology, liver comet assay, serum levels of aspartate aminotransferase (AST), alanine aminotransferase (ALT), and alkaline phosphatase (AP), and gut microbiome composition due to its major role in the pathogenesis of liver diseases [19,20].

## 2. Materials and Methods

### 2.1. Animals and Treatments

All animal procedures were performed in compliance with the Directive 2010/63/EU and ARRIVE guidelines and approved by the Veterinary Directorate of the Ministry of Agriculture, Forestry and Water Management, Republic of Serbia, License number 323-07-06124/2019-05.

In total, 40 female rats of Wistar strain (*Rattus norvegicus*), 2.5 months old, weighting 280.79 ± 4.91 g, were used for the experiment. The animals were acclimated to 22 ± 1 °C and maintained under a 12-h light/dark period, with ad libitum access to tap water and commercial standard rat food (Veterinary Institute, Subotica, Republic of Serbia). The rats were randomly divided into five groups, with eight rats per group, and housed two per cage.

The control group rats (average body mass 270.50 ± 5.18 g) were fed for four months with commercial standard rat food, with free access to tap water (Table 1).

The rats treated with thioacetamide (TAA group, average body mass 282.00 ± 5.62 g) were fed for four months with commercial standard rat food, with free access to tap water in which thioacetamide was dissolved in 300 mg/L concentration (Table 1).

The rats treated with thioacetamide and virgin olive oil (TAA+O group, average body mass 281.63 ± 11.08 g) were fed for four months with commercial standard rat food enriched with the VOO (10% mass/mass concentration), with free access to tap water in which thioacetamide was dissolved (300 mg/L) (Table 1).

The rats treated with thioacetamide and a low fullerene dose (TAA+F1 group, average body mass 296.50 ± 11.02 g) were fed for four months with commercial standard rat food enriched with the VOO (10% mass/mass concentration) in which C60 fullerene was dissolved in 0.2 mg/mL concentration, with free access to tap water in which thioacetamide was dissolved (300 mg/L) (Table 1).

The rats of the thioacetamide and a high fullerene dose group (TAA+F2 group, average body mass 271 ± 5.35 g) were fed for four months with commercial standard rat food enriched with the VOO (10% mass/mass concentration) in which C60 fullerene was dissolved in 1 mg/mL concentration, with free access to tap water in which thioacetamide was dissolved (300 mg/L) (Table 1).

TAA was obtained by Sigma-Aldrich, St. Louis, MO, USA, CAS Number 62-55-5, reagent grade 98%. Virgin olive oil was a commercially available oil (Olitalia, Italy). C60 fullerene was obtained by Bucky USA, Lake Jackson, TX, USA, CAS# 99685-96-8, 99% purity. Fullerene doses were selected to reach animal daily C60 intake of about 1 mg/kg b.m. and 5 mg/kg b.m. [8]. C60 was stirred in VOO for one week at room temperature due to high insolubility, covered with aluminium foil to avoid UV irradiation [10]. After one week of stirring, a cherry-red colour typical for C60-saturated vegetable oils was formed [21]. The food with VOO and/or VOO+C60 was prepared weekly. 

### 2.2. Sample Preparation

The body mass, food, and water intake were measured weekly during the whole experiment. At the end of the experiment, the animals were killed by decapitation using the Harvard guillotine and serum, and liver samples were collected and stored at −80 °C until the analysis.

Stool samples were freshly collected in a sterile plastic cup, immediately frozen at −80 °C, and kept until the gut bacteria composition was analysed.

### 2.3. Tissue Histology Analysis

Specimens of the liver tissues were paraffin-embedded and cut on 4 μm thick sections for haematoxylin and eosin (H&E), Masson-trichrome, and periodic acid-schiff (PAS) staining. Masson-trichrome staining was used to highlight fibrotic changes, while PAS accentuates glycogen accumulation. The tissue sections were examined under a light microscope (Olympus BX53, Hamburg, Germany) by two independent pathologists, and evaluated in respect to parenchymal injury (glycogen depletion, fatty change, ballooning degeneration, and necrosis), fibrosis, biliary injury (cholestasis, biliary hyperplasia, and CCC), and inflammation (portal space, inflammatory cells infiltration, and Kupffer cell hyperplasia), The scoring was as following: Glycogen depletion, 0—absent, 1—present; Fatty change, 0—absent, 1—focal, 2—diffuse; Ballooning degeneration, 0—absent, 1—present; Necrosis, 0—absent, 1—focal, 2—multifocal, 3—extensive; Fibrosis, 0—absent, 1—present; necrosis, 0—absent, 1—periportal fibrosis, 2—rare porto-portal septae, 3—numerous porto-portal septae, 4—cirrhosis; Cholestasis, 0—absent, 1—present; Biliary hyperplasia, 0—absent, 1—unifocal, 2—multifocal; CCC, 0—absent, 1—present; Portal space, 0—normal, 1—expanded; Inflammatory cells infiltration, 0—absent, 1—mild, 2—moderate, 3—severe; Kupffer cell hyperplasia, 0—absent, 1—present.

### 2.4. Oxidative Status Analysis

Liver samples were homogenized in 1:4 mass/volume cold phosphate-buffered saline using IKA T 10 Basic Ultra Turrax Homogenizer (IKA®-Werke GmbH & Co. KG, Staufen, Germany). After centrifugation (13,000× *g* for 30 min at 4 °C, Eppendorf microcentrifuge 5417R), the supernatant was collected and used for further analyses. All assays were done in triplicate.

Total SOD activity was measured by the method based on the SOD capacity to inhibit autoxidation of adrenaline to adrenochrome [22]. The reaction was performed in the incubation mixture containing 0.05 M Na_2_CO_3_, 0.1 M EDTA, pH 10.2, and monitored at 480 nm and 26 °C spectrophotometrically. One unit of SOD activity is defined as the amount of enzyme that caused 50% inhibition of the autoxidation of adrenaline under specified conditions. The results were expressed as specific activity of the enzyme in U/g tissue.

Catalase (CAT) activity was measured by the method of Clairborne [23]. A decrease in absorbance at 240 nm due to H_2_O_2_ decomposition was proportional to the enzyme activity. CAT activity was calculated using the millimolar extinction coefficient of H_2_O_2_ at 240 nm (0.0436 mM^−1^ cm^−1^) and expressed as U/mg of tissue.

GSH content was determined using Ellman’s method [24]. In the reaction of thiols with the chromogenic DTNB (5,5′-dithiobis-2-nitrobenzoate), the yellow dianion of 5-thio-2-nitrobenzoic acid (TNB) was formed. Absorbance was measured at 405 nm with a WALLAC 1420 Victor Multilabel Counter, LKB (London, UK). Quantification was done according to the GSH standard solutions (100–1000 μM). The final results were expressed as µmol/g of tissue.

GPx activity was measured by the method of Paglia and Valentine [25]. GPx catalyses the reduction of H_2_O_2_ using GSH as a source of an electron, which is oxidized to glutathione disulphide (GSSG). Newly formed GSSG is reduced back to GSH by GR, using nicotinamide adenine dinucleotide phosphate (NADPH) as an electron donor. GR activity was determined according to the method of Halliwell and Foyer [26]. The assay is based on the reduction of GSSG by NADPH in the presence of GR. The GPx and GR activities were measured by the decrease in absorbance at 340 nm caused by the oxidation of NADPH, calculated using the millimolar extinction coefficient of NADPH 6.22 mM^−1^ cm^−1^ at 340 nm and expressed as U/g of tissue. 

GST activity was assessed according to the method of Habig et al. [27]. The enzyme was assayed by its ability to conjugate GSH and 1-chloro-2,4-dinitrobenzene (CDNB). The increase of absorbance at 340 nm is directly proportional to the GST activity. The activity was calculated using an extinction coefficient of 9.6 mM^−1^ cm^−1^ and expressed as U/g of tissue.

The level of 4-HNE was determined according to the modified method of Gerard-Monnier et al. [28]. Briefly, standards (dilution series of 10 mM 1,1,3,3,″-tetramethoxypropane), blank (acetonitrile:methanol in 3:1 ratio) and samples (47 μL) were mixed with the working solution (152 µL). After gentle vortexing, methane sulfonic acid (35 μL) was added to determine 4-HNE. The mixtures were vortexed, heated at 45 °C/60 min, and then centrifuged (13,000× *g*, 15 min, 4 °C). The 580 nm absorbance in the supernatants was measured in a microplate reader (WALLAC 1420-Victor2 Multilabel Counter, PerkinElmer, Waltham, MA, USA), and the concentrations determined using the corresponding standard curves.

### 2.5. Western Immunoblotting 

#### 2.5.1. Preparation of the Whole Liver Homogenates and Liver Nuclear Protein Fractions 

For whole homogenate preparation, frozen liver samples were homogenized in a 1:4 mass/volume of ice-cold homogenization buffer (250 mM sucrose, 10 mM Tris-HCl, pH 7.6, 1 mM EDTA) supplemented with 1× phosphatase inhibitor Mix I, and 1× protease inhibitor Mix G (SERVA Electrophoresis GmbH, Heidelberg, Germany). The homogenates were filtered through gauze, centrifuged at 9700× *g* for 20 min at 4 °C, and the resulting supernatants were aliquoted, snap-frozen in liquid nitrogen, and stored at −80 °C.

For the preparation of nuclear protein extracts, samples of rat liver tissue were homogenized in a 1:4 mass/volume of ice-cold homogenization buffer (20 mM Tris-HCl pH 7.0, 1 mM EDTA, 10% glycerol, 50 mM NaCl, 2 mM DTT, 20 mM Na-phosphate buffer, protease, and phosphatase inhibitors). The homogenates were filtered through gauze and centrifuged at 3000× *g* for 15 min at 4 °C. The resulting pellets were further processed to generate nuclear protein fractions. The pellets were washed twice in HEPES buffer (25 mM HEPES pH 7.6, 1 mM EDTA, 1 mM EGTA, 10% glycerol, 50 mM NaCl, 2 mM DTT, protease, and phosphatase inhibitors). This was followed by centrifugation at 4000× *g* for 15 min at 4 °C, resuspension of the resulting pellets in a 1:1 mass/volume buffer containing 25 mM HEPES pH 7.6, 1 M Urea, 300 mM NaCl, 1% NP-40, protease and phosphatase inhibitors, and incubation of the suspensions on ice for 1 h with continuous agitation and frequent vortexing. The supernatant containing nuclear proteins was collected by centrifugation at 14,000× *g* for 10 min at 4 °C. Aliquoted samples were stored at −80 °C until use.

#### 2.5.2. Western Immunoblot Analysis

Protein samples of whole liver homogenates (30 µg) or nuclear extracts (25 µg) were separated by 12% SDS-PAGE and transferred onto polyvinylidene difluoride (PVDF) membranes (Hybond-P, Amersham Pharmacia Biotech, Little Chalfont, UK). The membranes were blocked in 5% non-fat condensed milk in Tris-buffered saline containing 0.2% Tween 20 (TBST) for 1 h at room temperature and then incubated overnight at 4 °C with polyclonal antibodies for HMGB1 (ab 18256), phospho-NF-ĸB p65 (Ser 311, ab 194926), NF-ĸB p65 (ab 7970), Nrf2 (ab 92946), HO-1 (ab 13243), β-actin (ab 8227), MnSOD/SOD-2 (sc 30080) and CuZnSOD/SOD 1 (sc-8637). PVDF membranes were washed with TBST and then incubated with horseradish peroxidase-conjugated secondary antibodies (ab 97051 or sc-2020) for 1.5 h at room temperature. Detection of immunoreactive bands was performed by an enhanced chemiluminescence detection system (Santa Cruz Biotechnology, Dallas, TX, USA) using an X-ray film (blots with HMGB1) or iBright CL1500 Imaging System (Thermo Fisher Scientific, Waltham, MA, USA). For re-probing, membranes were incubated in 2% SDS, 100 mM β-mercaptoethanol, and 62.5 mM Tris-HCl pH 6.8 for 35 min at 50 °C, then rinsed three times, blocked, and probed again with another antibody. The quantification of immunoreactive bands was performed using the TotalLab (Phoretix, Newcastle Upon Tyne, UK) electrophoresis software (version 1.10). For the Nrf2/ NF-κB p65 ratio determination, the same membrane for Western immunoblotting was used, with anti-Nrf2 antibody first (2 h, room temperature) and then, with anti-NF-κB p65 antibody (overnight, 4 °C).

### 2.6. Biochemical Analysis

After 16 h of overnight fast [29], weekly glycemia was measured by the Wellion CALLA Light blood glucose test strips system, using fresh tail capillary blood samples.

AST, ALT, and AP activities were measured in serum by the Roche Cobas C501 Chemistry analyser, using ALTL, ASTL, and ALP2L reagent cassettes.

The quantification of C60 in liver samples was performed on a CAMAG TLC Scanner equipped with Linomat 4. Tissue samples (50 mg) were dissolved in 1 mL of toluene and vortexed for 30 s. After that, the Eppendorf vials were centrifugated at 12,000× *g* for 20 min at 4 °C. The supernatant was transferred to the appropriate vials and kept in a freezer until analysis. The 5 µL of the analyte extract was applied to the 20 × 10 cm HPTLC silica gel plates (Art. 105641, Merck, Darmstadt, Germany) as a 6 mm band by using an Automatic TLC sampler 4 (ATS4, CAMAG, Muttenz, Switzerland). Ascending thin-layer chromatography was performed in a Cammag twin trough chamber using binary mobile phase: n-hexane: toluene (90:10, *v*/*v*). The solvent front was 90 mm. Before analysis, the chambers were saturated for 30 min. The measurement of fullerene was performed in Camag TLC Scanner 3 at 254 nm. The calibration curve was obtained by applying 1 µL, 2 µL, 3 µL, 4 µL and 5 µL of the fullerene standard solution (c = 1000 ppm). The linearity of the method was determined by ordinary least square analysis. Masses of C60 in samples were calculated according to the linear formula determined from the standard curve and recalculated to the amount of C60 (µg) per 1 mg of tissue.

### 2.7. Comet Assay

Liver comet assay was performed according to Tice et al. [30], with the single-cell liver suspension prepared as described by Wilson et al. [31].

### 2.8. Gut Microbiome Analysis

Extraction of ultra-pure gDNA from four faeces samples (approximately 200 mg each) was done using the ZymoBIOMICS™ DNA Miniprep Kit (D4304, Zymo Research Corporation, Irvine, CA, USA). Total DNA was isolated and purified following the manufacturer’s protocol. The samples were commercially analysed by FISABIO Sequencing and Bioinformatics Service, Center for Public Health Research, Valencia, Spain, using the following Next Generation Sequencing order: lllumina16S V3-V4 amplicon library preparation, MiSeq 300 bp PE sequencing, and 100,000 reads per sample.

### 2.9. Statistical Analysis

Where appropriate, some of the results were graphically expressed as time-course curves, which were subsequently recalculated into the values of the area under the curve (AUC), and presented as % of control. All data were presented as mean ± standard error. The differences in investigated parameters between the groups were calculated in SIGMAPLOT using one-way ANOVA. When significant differences were found, pairwise comparisons were performed using Holm-Sidak post hoc tests. The level of significance was defined as *p* < 0.05.

The metagenomic analysis was performed in R 3.6.1 [32], following the dada2 (version 1.12.1) pipeline [33]. Prior to performing sequence analysis, primer sequences were removed using BBDuk software (https://jgi.doe.gov/data-and-tools/bbtools, accessed on 30 April 2019) with kmer length 15 and Hamming distance 1 (1 mismatch was allowed). After primer removal, sequences were right trimmed to 250 bases for the forward reads and 200 bases for reverse reads, based on the distribution of fastq quality scores. After truncation, reads with higher than four expected errors (calculated as sum(10^(-Q/10))—where Q is the quality score) for forward reads and reverse reads were all discarded. After dada2 error estimation and sequence inference, sequence pairs were merged using a minimum overlap of 20 bases without mismatches, and all sequences shorter than 385 and longer than 430 nucleotides were discarded based on the sequence length distribution. Finally, chimeric sequences were removed using default parameters in dada2, resulting in ~65% of the initial sequenced reads being retained. Taxonomy assignment up to genus level was performed using the Silva v132 (https://www.arb-silva.de/documentation/release-132/, accessed on 7 March 2019) database with the RDP Naive Bayesian Classifier algorithm [34] as implemented in dada2, using kmer size 8 and 100 bootstrap replicates. The minimum bootstrap confidence for assigning a taxonomic level was set to 50.

## 3. Results and Discussion

**Figure 1:**
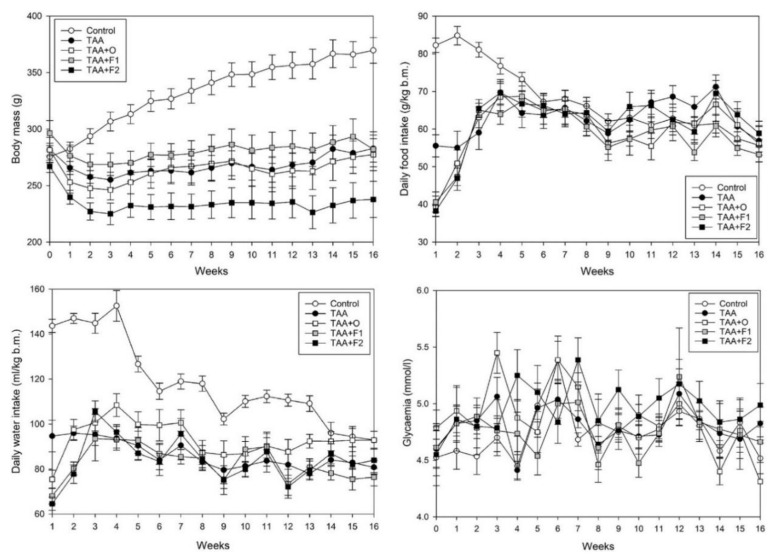
Average weekly body mass gain (g/100 g b.m.), daily food (g/kg b.m.) and water (mL/kg b.m.) intake, as well as glycaemia (mmol/L) in control, and rats treated with thioacetamide (TAA), thioacetamide + virgin olive oil (TAA+O), thioacetamide + C60 fullerene lower dose (TAA+F1), and thioacetamide + C60 fullerene higher dose (TAA+F2). The number of animals per group = 8.

Data on changes in animal body weight, food, water, VOO and fullerene intake, as well as changes in glycaemia, are shown in Figure 1 and Table 2. As can be seen in Figure 1, control animals’ body masses increased in respect to the initial values at the beginning of the experiment (point zero on the corresponding graph in Figure 1), while food and water intake decreased. In the remaining groups, the food and water intake during the first few weeks was significantly lower compared to controls, which led to weight gain stagnation in these animals. The results presented as an area under the curve (AUC) show a decrease of about 20–25% in body mass, 10–15% in food consumption, and 20–35% in water consumption relative to controls (Table 2). There were no significant differences in listed parameters between the TAA, TAA+O, and TAA+F1 groups, so it can be concluded that observed changes are the result of TAA-toxicity, on which neither VOO nor a lower dose of C60 had a protective effect. A significant decrease in body mass gain and water intake occurred only in the TAA+F2 group (Table 2), indicating the detrimental effect of a higher C60 dose.

At the beginning of the experiment (point zero on the corresponding graph in Figure 1), the average serum glycaemia was 4.53 ± 0.16 mmol/L in control, 4.60 ± 0.16 mmol/L in TAA, 4.79 ± 0.16 mmol/L in TAA+O, 4.61 ± 0.18 mmol/L in TAA+F1, and 4.55 ± 0.28 mmol/L in the TAA+F2 group, without significant differences between groups. As can be seen in Figure 1 and Table 2, there were no significant changes in glycaemia between TAA, TAA+O, and TAA+F1 groups, indicating preserved glucose homeostasis. Glycaemia slightly increased only in the TAA+F2 group, which is another sign of the harmful effect of a high dose of fullerene. Previously we reported similar effects of a higher C60 dose, hypothesizing that they are caused by its pro-oxidant behaviour [35]. It is known that in an aqueous milieu, such as blood and/or cytoplasm, C60 becomes prone to self-aggregation [36] and, consequently, to pro-oxidant action [37,38]. Therefore, it is possible that negative effects of a higher dose of fullerene on body mass, water intake, and glucose homeostasis are all consequences of its enhanced self-aggregation.

There were no significant differences in VOO intake between rats of the TAA+O, TAA+F1, and TAA+F2 groups (Table 2). These values, calculated to estimate the human equivalent dose [39], result as an average daily human consumption of about 60 g. This is an important fact since a diet with high amounts of otherwise healthy oils can lead to liver lipid homeostasis disorders [40]. C60 intake was around 1.15 mg/kg b.m./day in rats of the TAA+F1 group and 5.23 mg/kg b.m./day in rats of the TAA+F2 group, confirming that the preferred 1:5 ratio between the low and high fullerene doses was achieved. It should be noted that the 5-fold higher dose of C60 led to only a 60% liver bioaccumulation increase in respect to its lower dose (Table 2), indicating the limit of fullerene bioabsorption.

**Figure 2:**
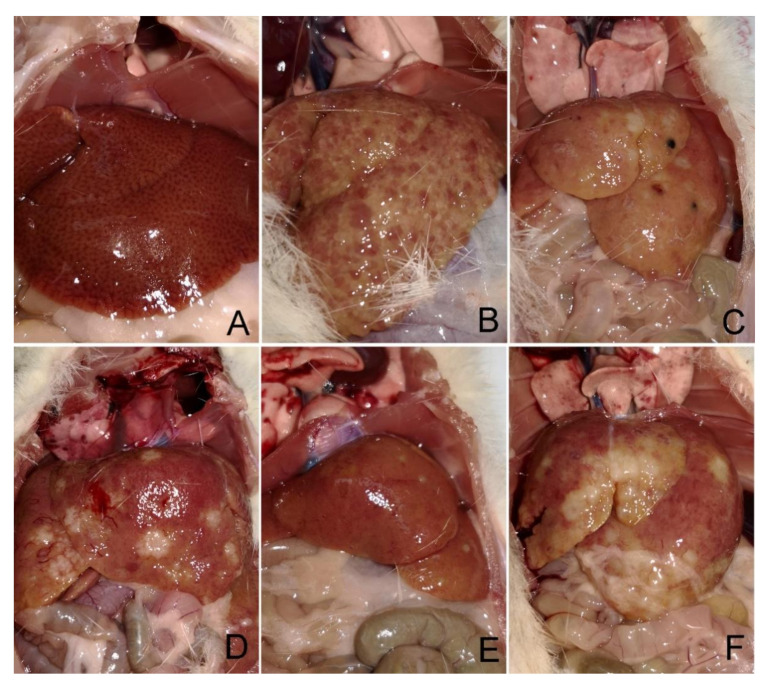
Representative photographs of liver appearances during the organ isolation in control, and rats treated with thioacetamide (TAA), thioacetamide + virgin olive oil (TAA+O), thioacetamide + C60 fullerene lower dose (TAA+F1), and thioacetamide + C60 fullerene higher dose (TAA+F2). (**A**) the liver of rats from the control group; (**B**,**C**) the liver of rats from the TAA group; (**D**) the liver of rats from the TAA+O group; (**E**) the liver of rats from TAA+F1 group; (**F**) the liver of rats from TAA+F2 group.

The TAA model is widely recognised as a reliable method for induction of both hepatocellular (HCC) and cholangiocellular carcinoma (CCC) [41,42], which is why liver histology was applied in our study. However, it was possible to notice a difference between treatments by a visual observation even during liver isolation (Figure 2).

The most serious histological changes observed after administration of thioacetamide were the induction of fibrosis and development of CCC in the rats of all TAA-supplemented groups. CCC was most common in the TAA+O group, in which all 8/8 animals developed cancer. In TAA and TAA+F2 groups, CCC was observed in 75% (6/8) of animals, while only 50% (4/8) of animals developed it in the TAA+F1 group. HCC was not observed in all experimental groups, but it should be noted that it is considered as a multi-step disease, in which carcinoma development is preceded by fibrosis and cirrhosis [43]. In a study conducted by Yeh et al., invasive CCC preceded cirrhosis by at least 4 weeks [44]. The fact that Stage IV fibrosis developed in 50% of animals from all experimental groups excluding controls suggests that TAA may induce HCC only with the underlying cirrhosis.

**Figure 3:**
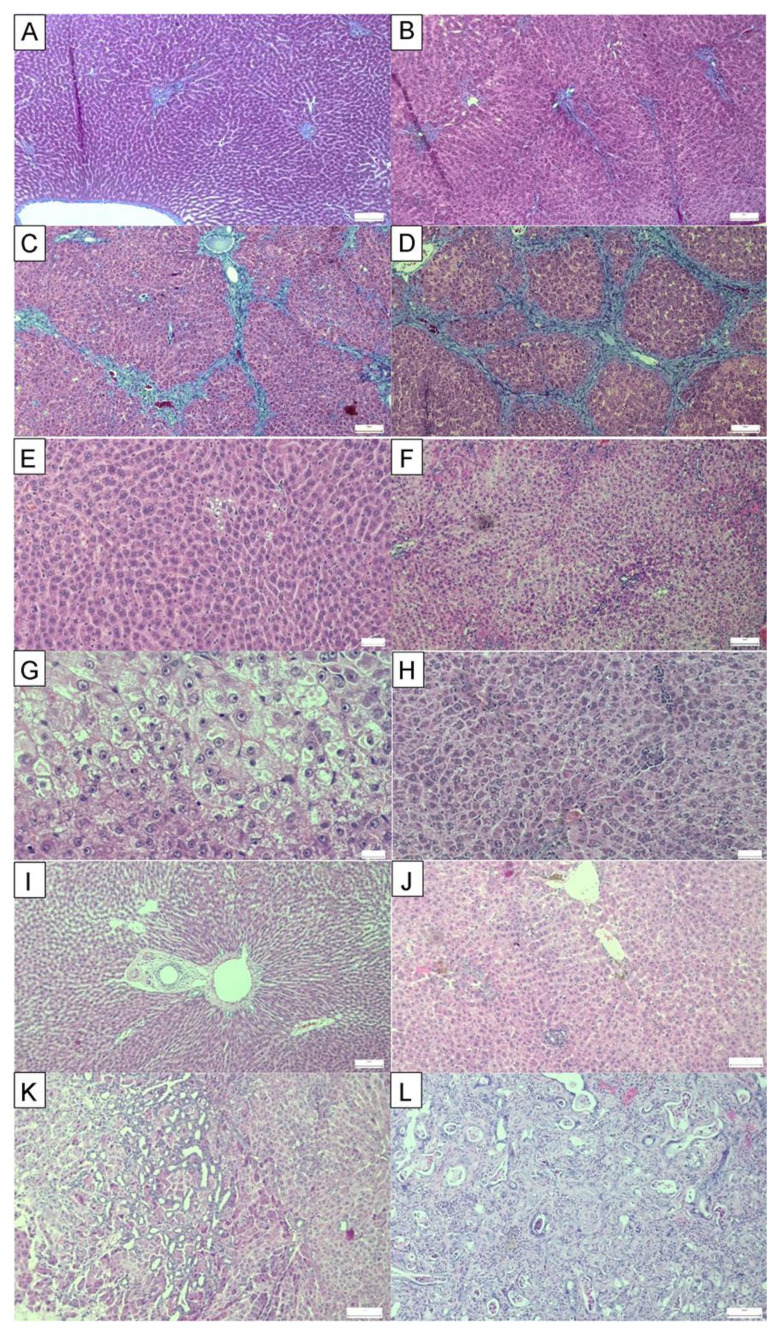
Representative micrographs of liver histology in control, and rats treated with thioacetamide (TAA), thioacetamide + virgin olive oil (TAA+O), thioacetamide + C60 fullerene lower dose (TAA+F1), and thioacetamide + C60 fullerene higher dose (TAA+F2). Fibrosis (Masson-trichrome staining): (**A**) liver without fibrosis in the control group, (**B**) mild periportal fibrotic expansion in TAA+F1 group, (**C**) bridging fibrosis in TAA group, and (**D**) cirrhosis in TAA+O group; Parenchymal injury (PAS staining): (**E**) small focus of fatty change in group TAA+F1, (**F**) more severe fatty change in TAA group, (**G**) ballooning degeneration in group TAA+F2, and (**H**) focal necrosis and fatty change in TAA+O group; Biliary injury (H&E staining): (**I**) normal morphology in the control group; (**J**) biliary pigment in TAA group; (**K**) biliary hyperplasia in TAA+O group; (**L**) cholangiocellular carcinoma in TAA+F1 group.

Parenchymal injury, fibrosis, and biliary injury and inflammation were also more prominent in the TAA+O group than all other experimental groups (Figure 3 and Table 3). Further, analysing individual parameters of parenchymal damage, we observed more pronounced fatty accumulation in the liver of rats of the TAA+O group in respect to the TAA group. It should be noted that our results are in accordance with the literature data showing the detrimental hepatic effect of VOO [45]. For example, Kouka et al., showed that VOO, especially when rich in polyphenolic content, induced oxidative stress in a rat’s liver [46]. Parenchymal injury, biliary changes, fibrosis, and inflammation were less severe in rats of the TAA+F1 and TAA+F2 groups than the TAA+O group, although the stronger hepatoprotective effect was noticed with a lower dose of fullerene. However, some of these changes were more severe in the liver of TAA+F1 and TAA+F2 rat groups than the TAA group, which, considering the above mentioned, could be explained by the detrimental effect of VOO. Taken all together, the results of liver histology suggest that VOO may enhance toxic effects of TAA and that fullerene is more protective of the liver when applied in lower doses.

**Figure 4:**
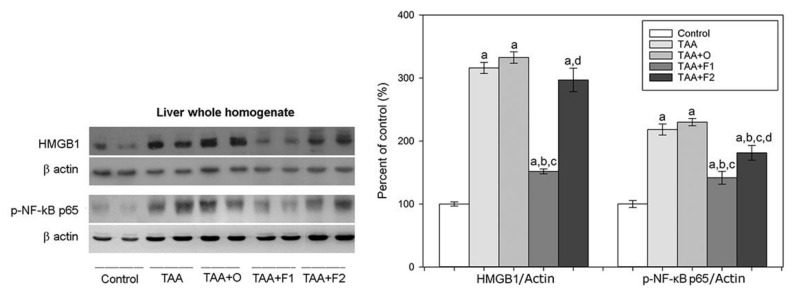
Protein expression levels of HMGB1 and phospo-NF-kB p65 in whole liver homogenates in control, and rats treated with thioacetamide (TAA), thioacetamide + virgin olive oil (TAA+O), thioacetamide + C60 fullerene lower dose (TAA+F1), and thioacetamide + C60 fullerene higher dose (TAA+F2). Data are given as mean ± standard error. Minimal significance level: *p* < 0.05. Significantly different: ^a^ in respect to Control; ^b^ in respect to TAA; ^c^ in respect to TAA+O, and ^d^ in respect to TAA+F1 group. The number of animals per group = 8.

To examine whether C60 has an anti-necrotic role in TAA-induced liver injured, we investigated the expression level of HMGB1 in the whole liver homogenates. HMGB1, a member of the damage-associated molecular patterns (DAMPs) molecule group, is released passively from damaged, dying or stressed cells, making it a necrotic marker [47]. Additionally, released HMGB1 exerts a cytokine-like effect on immune and non-immune cells by promoting NF-κB p65 downstream signalling and induction of proinflammatory cytokines [48]. Our results showed that in comparison with the control group, treatment with TAA (alone or in combination with VOO) significantly increased the liver’s whole homogenate level of HMGB1 expression (3.2- and 3.3-fold, respectively), and consequently the level of phosphorylated NF-κB p65 (2.2- and 2-3-fold, respectively) (Figure 4). In comparison to the TAA+O rat group, in the TAA+F1 group, the expression level of HMGB1 and p-NF-κB p65 decreased by about 54% and 39%, respectively, while in TAA+F2-treated rats, only p-NF-κB p65 expression level decreased by about 22% (Supplementary images). These results suggested that only a lower dose of C60 fullerene has the potential to inhibit activation of NF-κB p65 and, consequently, necrotic events in TAA-induced liver injury. According to a study by Ge et al., HMGB1/NF-κB p65 signalling also exert an important effect on hepatic fibrosis via activation of a class of small non-coding RNA (miR-146b), which promotes hepatic stellate cells (HSCs) activation and proliferation [49]. Based on these findings and fibrotic changes observed by the liver histology (Figure 2), it is reasonable to conclude that C60 fullerene, especially in a lower dose, may decrease the degree of liver fibrosis through the HMGB1/NF-κB p65 signalling-pathway regulation.

Oxidative stress is known to have an important role in the pathogenesis of liver diseases [50,51]. Cytochrome P450 isoform P4502E1 (CYP2E1) is the major source of hepatic ROS generation [52], being also responsible for the production of the TAA reactive metabolites, S-oxide, and S,S-dioxide. During TAA-metabolism, dioxygen is reduced to superoxide radical by flavin-containing monooxygenase and cytochrome P450 and then catalysed to hydrogen peroxide. However, hydrogen peroxide is a source of the highly reactive hydroxyl radical, responsible for enhanced lipid peroxidation [53]. As shown in Table 4, total SOD and GPx activities increased, while CAT activity decreased significantly in rats that received TAA and TAA+O compared to the controls. 

Catalase and GPx are known to cooperate in H_2_O_2_ catabolism, complementing each other [54]. Therefore, the increased GPx activity could be the compensatory mechanism of the lowered CAT activity [50], but also a sign of increased H_2_O_2_ production, as has been seen in activated hepatic stellate cells [55]. The GPx relies on the steady supply of GSH, using it as the reducing substrate in its peroxidase activity [56]. However, once the GPx peroxidase activity has been accomplished, it is up to the GR to re-reduced oxidised glutathione to the GSH [57]. Therefore, the increased GR activity level and the decreased liver glutathione content in TAA and TAA+O groups could be a consequence of simultaneous GPx activity increase in the same groups of animals (Table 4). Finally, TAA treatment also increased GST activity, with the effect being more prominent in the liver of rats from the TAA+O group than in the TAA group (Table 4). Glutathione S-transferases are a family of phase II metabolic isozymes which catalyse the conjugation of xenobiotic substrates with the reduced form of glutathione for the purpose of detoxification. Thus, the GST activity increase in TAA- and TAA+O-treated animals could also participate in the GSH liver content decrease, and the GR activity increase. Based on all aforementioned, it is evident that TAA caused hepatic oxidative stress, in consistence with the literature data showing a significant decrease in the levels of liver CAT and GSH [58,59], and the increased SOD activity after TAA treatment [60]. The explanation of why some of these effects were more prominent in the group receiving VOO with the TAA could be that the excessive supplementation with vegetable oils could cause liver steatosis [40], causing increased oxidative stress [61]. While administering both fullerene doses restored total SOD activity to control values, only the low C60 dose had the same effect on CAT activity (Table 4). A higher level of CAT activity indicates that fullerene could promote the liver antioxidative system (AOS), similarly to the previous findings on the role of quercetin in the prevention of TAA-induced liver injury [60]. Upon C60 treatment, both GR and GST activities and the GSH level remained unchanged compared to TAA intoxicated rats (Table 4). Intriguingly, the level of 4-HNE, one of the products of lipid peroxidation with a key role in stress-mediated signalling [62], remained unchanged in TAA and TAA+O rat groups in respect to controls, in contrast to both fullerene groups in which 4-HNE concentration was reduced.

**Figure 5:**
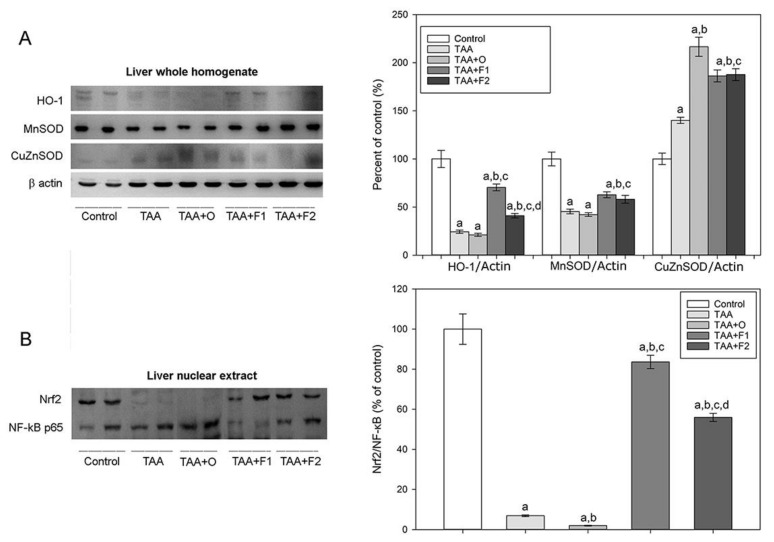
Protein expression levels of (**A**) haeme oxygenase-1 (HO-1), manganese superoxide-dismutase (MnSOD) and copper-zinc superoxide-dismutase (CuZnSOD) in whole liver homogenates, and (**B**) protein expression levels of total Nrf2 and NF-kB p65 in nuclear extracts in control, and rats treated with thioacetamide (TAA), thioacetamide + virgin olive oil (TAA+O), thioacetamide + C60 fullerene lower dose (TAA+F1), and thioacetamide + C60 fullerene higher dose (TAA+F2). Data are given as mean ± standard error. Minimal significance level: *p* < 0.05. Significantly different: ^a^ in respect to Control; ^b^ in respect to TAA; ^c^ in respect to TAA+O, and ^d^ in respect to TAA+F1 group. The number of animals per group = 8.

Results of the AOS enzymes’ activity analysis are in complete agreement with the results of analysis of HO-1, CuZnSOD, and MnSOD relative expression in whole liver homogenates (Figure 5A and Supplementary images). Our results demonstrated a significant decrease of HO-1 (75%) and MnSOD (55%) expression level in the liver of TAA- and TAA+O-treated animals in comparison to controls, while the expression of CuZnSOD increased 1.4-fold in TAA and 2.2-fold in TAA+O-treated animals. The supplementation with C60 fullerene lowered the expression level of CuZnSOD by about 20% independently of the dose applied and augmented the expression of HO-1 and MnSOD to reach about 70% and 60% of the control values in the case of lower, and 40% and 60% in the case of higher fullerene dose, respectively. As a result of CuZnSOD and MnSOD expression level interplay, the total SOD activity in the liver of TAA- and TAA+O-treated animals increased, and in the TAA+F1 and TAA+F2 groups decreased (Table 4).

Despite the detected increase in total SOD activity in the liver in both TAA groups, the results presented herein show that the relative expression level of CuZnSOD in TAA treated rats was increased, while the level of MnSOD was decreased. Considering that Nrf2 and NF-κB pathways interplay through a range of complex molecular interactions, and depending on the cell type and tissue [63], we determined the ratio between total Nrf2 and NF-κB p65 in the liver nuclear extracts (Figure 5B). In the liver of TAA and TAA+O-treated rats, nuclear expression of total NF-κB p65 increased, especially in TAA+O-treated rats (2.7-fold), while the nuclear expression of Nrf2 strongly decreased. As a result, the Nrf2/NF-κB p65 ratio was decreased in comparison to the control group, therefore reducing the efficiency of HO-1 and MnSOD gene expression in TAA and TAA+O-treated rats. Accordingly, a Nrf2/NF-κB p65 ratio increase in the liver nuclear extracts of TAA+F1- and TAA+F2-treated rats resulted in the increased expression of HO-1 and MnSOD. The rise in expression of CuZnSOD in TAA- and TAA+O -treated rats also could be interpreted as the result of increased nuclear NF-kB p65 expression since it is identified as a key mediator of the CuZnSOD gene regulation [64]. Finally, Nrf2 is known to control the expression of the catalytic and the modifier subunits of the glutamate-cysteine ligase complex that catalyses the reaction of glutamate with cysteine, the rate-limiting step in the synthesis of GSH [65], which could be an explanation for the decrease in GSH liver content seen in TAA- and TAA+O-treated rats (Table 4). Taken together, the presented findings demonstrated that C60 fullerene ameliorate TAA-induced liver injury via suppression of liver oxidative stress and enhancement of the antioxidant machinery through upregulating Nrf2 signal pathways and its downstream HO-1 and MnSOD expression.

Table 5 presents the results of serum AST, ALT, and AP activities, and liver comet assay. It can be seen that AST and ALT levels were increased in all experimental groups in comparison to controls. There were no significant differences in their activity between TAA-, TAA+O-, TAA+F1-, and TAA+F2-treated rats, indicating that the observed changes belong solely to the thioacetamide. However, in the case of AP, a 5-fold increase seen in the TAA group was further increased 2-fold in the TAA+O group and reduced only in the TAA+F1, but not in the TAA+F2-treated rats. It is known that elevated AP, out of proportion to AST and ALT, is typical for the cholestatic pattern of liver disease, i.e., bile duct obstruction, cholestasis, primary biliary cirrhosis, cystic fibrosis, and CCC [66], all belonging to exactly the same pathology previously described in the histology part (Figure 3 and Table 3). However, some non-hepatic sources of the AP activity increase cannot be excluded. For example, TAA causes tissue injury not only in the liver but also in the kidneys [67,68]. In addition, it is known that AP has an important anti-inflammatory role in the pathogenesis of gastrointestinal and systemic inflammation [69]. Intestinal AP deficiency has been associated with increased gastrointestinal inflammation [70], which could be reduced by therapeutic supplementation with AP [71]. Having this in mind, we assume that the TAA-induced rise in the AP activity, together with its drop in the TAA+F1-treated rats, could be the part of the same anti-inflammatory response to the increased inflammation in TAA-treated rats and decreased inflammation in the fullerene low dose-treated animals. As for the liver comet assay, it is clear that TAA causes strong hepatocellular DNA damage (4-fold increase in TAA group in respect to controls) (Table 5). Intriguingly, in this case, concurrent addition of VOO exerted a beneficial effect since the percentage of DNA in comet tail was almost two times lower in TAA+O than in the TAA-treated animals. Treatment with both fullerene doses was equally effective in the prevention of TAA-induced DNA damage, returning the values to control levels.

The relationship between the gut microbiome and liver diseases is well established [19]. For example, cirrhosis patients have general microbiota overgrowth in the small intestine, while selective bacterial sanitization with antibiotics could be beneficial for patients with decompensated cirrhosis. The gut microbiota is also responsible for bile acid deconjugation and conversion of primary bile acids into secondary, and cirrhotic patients usually have a lower abundance of bacteria capable of bile acid conversion [20]. In turn, bile acids act as ligands for the bile-acid-membrane TGR5 receptor and bile-acid-activated FXR receptor, both playing a pivotal role in lipid and glucose homeostasis and regulation of the gut permeability and inflammatory responses [72]. Considering that diet and different other stimuli affect the gut microbiome and induce its dysbiosis [73], we studied the effects of TAA and C60 on the composition of the caecal microbiota.

**Figure 6:**
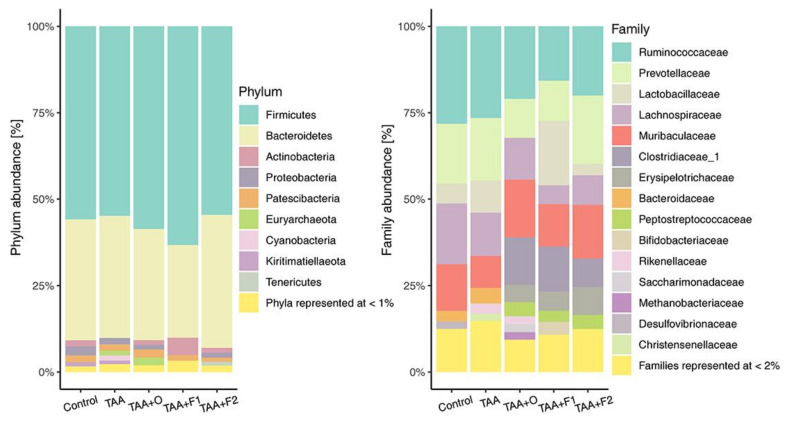
Percentage abundance on phylum (**left**) and family (**right**) level in control, and rats treated with thioacetamide (TAA), thioacetamide + virgin olive oil (TAA+O), thioacetamide + C60 fullerene lower dose (TAA+F1), and thioacetamide + C60 fullerene higher dose (TAA+F2).

The composition of gut microbiota at the phylum level is shown in Figure 6. As can be seen, two phyla, Firmicutes and Bacteroidetes, representing approximately 90% of the gut microbiota, are characterized with the inter-treatment changes that account for several percent, and that could be considered as not significant. However, these two phyla changes are observable across five bacterial families, three of them belonging to the phylum Firmicutes (*Lactobacillaceae, Clostridaceae*, and *Ruminococcaceae*), and two of them belonging to the phylum Bacteroidetes (*Prevotelaceae* and *Muribaculaceae*) (Figure 6).

A significant increase of *Lactobacilaceae* family abundance was observed in TAA+F1 treated rats, while a notable drop was observed in TAA+O and TAA+F2 rat groups. *Lactobacillaceae* is an extensively studied family because of its ability to promote host health through the production of bacteriocins that protect against pathogens [74]. As carbohydrate fermenting microorganisms, they produce lactic and other organic acids and consequently contribute to colonization and beneficial interactions between host and other microbiota constituents [75]. Families *Ruminococaceae* and *Clostridiaceae* are dietary polysaccharide-decomposing bacteria, producers of short-chain fatty acids, mainly acetate, propionate, and butyrate, that serve as an important source of energy [76], and regulators of intestinal homeostasis through anti-inflammatory actions [77]. Our results showed that *Ruminococaceae* abundance was decreased in the gut of TAA+O-, TAA+F1-, and TAA+F2-group rats, while *Clostridiaceae* abundance was strongly increased. The reduced Ruminococcus abundance has been reported in patients with ulcerative colitis and Crohn’s disease [78], while clostridia decrease seen in elderly people could be connected with some of their metabolic dysregulation and drop in immunity [79]. Regarding Bacterioidetes, in the gut of TAA+O- and TAA+F1-group rats, we found a decrease in the abundance of *Prevotellaceae*, bacteria known to exacerbate intestinal inflammation by the reduction of interleukin IL-18 production [80]. The abundance of *Muribaculaceae*, a bacteria known to negatively correlate with obesity-related diseases [81], was decreased in TAA, and increased in the gut of TAA+O, TAA+F1 and TAA+F2 groups. Based on all the above mentioned, it could be concluded that changes in the gut microbiota are not straightforward. For example, the *Lactobacillaceae* compromised abundance in TAA+O and TAA+F2 rat groups could explain the increased biliary injury and CCC occurrence seen in these animals (Figure 2 and Table 3). On the other hand, the Bacterioidetes abundance changes suggest a beneficial effect of VOO and both fullerene doses in respect to TAA treatment only. Finally, the physiological meaning of the *Ruminococaceae* and *Clostridiaceae* ratio reciprocal changes seen in the animals from the TAA+O, TAA+F1, and TAA+F2 groups remains to be clarified.

## 4. Conclusions

In line with the increased interest in the use of nanotechnology for biomedical applications, we investigated the effects of potent free-radical scavenger C60 fullerene in TAA-induced rat liver injury. The obtained data indicated the dose-dependent hepatoprotective effects of C60. In a low dose, C60 fullerene was more efficient in terms of liver antioxidative and anti-inflammatory protection and reduced tissue fibrosis, necrosis, and CCC development. The ability of C60 to upregulate Nrf2 and downregulate HMGB1/NF-kB p65 signalling pathways could contribute to its actions. Our data also implicated that VOO may enhance toxic effects of TAA, which is why the development of water-soluble C60 derivates could be a more promising approach in its therapeutic use.

## Figures and Tables

**Table 1:** The study designs. Control—control group of animals (free access to tap water and commercial standard rat food); TAA—thioacetamide group of animals (free access to commercial standard rat food, and tap water with the thioacetamide dissolved in 300 mg/L concentration); TAA+O—thioacetamide + virgin olive oil group of animals (free access to commercial standard rat food enriched with the oil in 10% mass/mass concentration, and tap water with the thioacetamide dissolved in 300 mg/L concentration); TAA+F1—thioacetamide + fullerene C60 low dose group of animals (free access to commercial standard rat food enriched with the oil in 10% mass/mass concentration in which C60 was dissolved in 0.2 mg/mL concentration, and tap water with the thioacetamide dissolved in 300 mg/L concentration); and TAA+F2—thioacetamide + fullerene C60 high dose group of animals (free access to commercial standard rat food enriched with the oil in 10% mass/mass concentration in which C60 was dissolved in 1 mg/mL concentration, and tap water with the thioacetamide dissolved in 300 mg/L concentration). The experiment lasted 4 months. The number of animals per group = 8.

Groups	Application of TAA	Application of VOO without C60	Application of VOO with Low C60 Dose	Application of VOO with High C60 Dose
Control	none	none	none	none
TAA	+	none	none	none
TAA+O	+	+	none	none
TAA+F1	+	none	+	none
TAA+F2	+	none	none	+

**Table 2:** The area under the curve (AUC, % of control) of the corresponding curves from Figure 1 (i.e., weekly body mass gain, daily food and water intake, and glycaemia), VOO (gr/kg b.m./day) and C60 fullerene (mg/kg b.m./day) average daily consumption during the whole experiment, and the liver C60 fullerene bioaccumulation (μg/g tissue) in control, and rats treated with thioacetamide (TAA), thioacetamide + virgin olive oil (TAA+O), thioacetamide + C60 fullerene lower dose (TAA+F1), and thioacetamide + C60 fullerene higher dose (TAA+F2). Data are given as mean ± standard error. Minimal significance level: *p* < 0.05.

Groups	Control	TAA	TAA+O	TAA+F1	TAA+F2
Body mass AUC	100.00 ± 1.16	80.36 ± 0.88 ^a^	78.80 ± 1.33 ^a^	85.17 ± 1.20 ^a^	74.56 ± 1.04 ^a,b,c,d^
Food intake AUC	100.00 ± 1.52	88.80 ± 1.77 ^a^	89.19 ± 2.17 ^a^	86.52 ± 1.53 ^a^	85.29 ± 1.66 ^a^
Water intake AUC	100.00 ± 1.74	78.70 ± 1.28 ^a^	77.77 ± 1.56 ^a^	75.03 ± 1.40 ^a^	66.81 ± 1.30 ^a,b,c,d^
Glycaemia AUC	100.00 ± 0.76	101.29 ± 0.83	101.37 ± 1.11	101.20 ± 1.20	107.30 ± 1.26
VOO intake	–	–	5.923 ± 0.127	5.805 ± 0.094	5.355 ± 0.115
C60 intake	–	–	–	1.146 ± 0.026	5.231 ± 0.126 ^d^
C60 liver bioaccumulation	–	–	–	21.04 ± 0.60	33.32 ± 0.98 ^d^

Significantly different: ^a^ in respect to Control; ^b^ in respect to TAA; ^c^ in respect to TAA+O, and ^d^ in respect to TAA+F1 group. The number of animals per group = 8.

**Table 3:** Liver histology score system. Average values in control, and rats treated with thioacetamide (TAA), thioacetamide + virgin olive oil (TAA+O), thioacetamide + C60 fullerene lower dose (TAA+F1), and thioacetamide + C60 fullerene higher dose (TAA+F2).

Groups	Control	TAA	TAA+O	TAA+F1	TAA+F2
Parenchymal injury	0.15	1.550	2.025	1.525	1.675
Fatty Change	0.25	1.250	2.375	1.500	1.875
Fibrosis	0	3.625	3.750	2.500	3.000
Biliary injury	0	0.662	0.850	0.525	0.775
Cholangiocarcinoma	0	75%	100%	50%	75%
Inflammation	0	0.844	0.900	0.731	0.794

**Table 4:** Liver activities of total superoxide dismutase (SOD, U/mg tissue), catalase (CAT, U/mg tissue), glutathione peroxidase (GPx, U/g tissue), glutathione reductase (GR, U/g tissue) and glutathione S-transferase (GST, U/g tissue), as well as glutathione (GSH, μmol/g tissue), and 4-hydroxynonenal content (4-HNE, μmol/L) in control, and rats treated with thioacetamide (TAA), thioacetamide + virgin olive oil (TAA+O), thioacetamide + C60 fullerene lower dose (TAA+F1), and thioacetamide + C60 fullerene higher dose (TAA+F2). The data are given as mean ± standard error. Data are given as mean ± standard error. Minimal significance level: *p* < 0.05.

Groups	Control	TAA	TAA+O	TAA+F1	TAA+F2
SOD	6.355 ± 0.320	8.623 ± 0.506 ^a^	11.623 ± 0.388 ^a,b^	6.049 ± 0.203 ^b,c^	5.970 ± 0.449 ^b,c^
CAT	71.815 ± 1.995	54.900 ± 2.614 ^a^	56.399 ± 4.623 ^a^	92.255 ± 2.352 ^a,b,c^	62.124 ± 3.931 ^d^
GPx	37.167 ± 1.846	48.911 ± 2.769 ^a^	48.265 ± 1.424 ^a^	35.263 ± 1.851 ^b,c^	29.688 ± 2.004 ^b,c^
GR	24.963 ± 1.686	39.859 ± 0.867 ^a^	44.164 ± 2.041 ^a^	45.784 ± 2.075 ^a^	46.939 ± 2.613 ^a^
GST	162.477 ± 11.425	325.490 ± 20.809 ^a^	549.839 ± 30.678 ^a,b^	550.760 ± 35.467 ^a,b^	516.204 ± 32.408 ^a,b^
GSH	37.938 ± 1.460	29.553 ± 1.227 ^a^	31.094 ± 1.351 ^a^	31.155 ± 1.222 ^a^	30.205 ± 2.228 ^a^
HNE	1.865 ± 0.126	1.757 ± 0.167	1.567 ± 0.133	1.421 ± 0.143 ^a^	1.391 ± 0.176 ^a^

Significantly different: ^a^ in respect to Control; ^b^ in respect to TAA; ^c^ in respect to TAA+O, and ^d^ in respect to TAA+F1 group. The number of animals per group = 8.

**Table 5:** The serum activity levels (U/L) of aspartate aminotransferase (AST), alanine aminotransferase (ALT) and alkaline phosphatase (AP), and a liver comet assay (% of DNA in comet tail) in control, and rats treated with thioacetamide (TAA), thioacetamide + virgin olive oil (TAA+O), thioacetamide + C60 fullerene lower dose (TAA+F1), and thioacetamide + C60 fullerene higher dose (TAA+F2). Data are given as mean ± standard error. Minimal significance level: *p* < 0.05.

Groups	Control	TAA	TAA+O	TAA+F1	TAA+F2
AST	177.38 ± 3.59	207.38 ± 5.91 ^a^	200.88 ± 4.43 ^a^	212.89 ± 4.22 ^a^	216.25 ± 6.19 ^a^
ALT	36.20 ± 2.19	53.25 ± 2.46 ^a^	52.75 ± 3.14 ^a^	59.88 ± 2.39 ^a^	61.88 ± 3.17 ^a^
AP	30.38 ± 2.04	164.50 ± 13.08 ^a^	302.50 ± 7.41 ^a,b^	262.38 ± 8.20 ^a,b,c^	300.75 ± 6.49 ^a,b,d^
Comet	2.754 ± 0.282	9.274 ± 0.717 ^a^	5.562 ± 0.392 ^a,b^	2.072 ± 0.137 ^b,c^	2.972 ± 0.220 ^b,c^

Significantly different: ^a^ in respect to Control; ^b^ in respect to TAA; ^c^ in respect to TAA+O, and ^d^ in respect to TAA+F1 group. The number of animals per group = 8.

## Data Availability

All study data are included in the article and Supplementary Materials.

## Supplementary Materials

The original images of Western blots are available online at https://www.mdpi.com/article/10.3390/antiox10060911/s1.
