# Supplementary material for: Effects of C60 Fullerene on Thioacetamide-Induced Rat Liver Toxicity and Gut Microbiome Changes
Source: Antioxidants (Basel). 2021 Jun 4;10(6):911. doi: 10.3390/antiox10060911 (PMC8226855; doi:10.3390/antiox10060911)

1. Original images of Western blot used in Figure 4. Protein expression levels of HMGB1 in whole liver homogenates in control, and rats treated with thioacetamide (TAA), thioacetamide + virgin olive oil (TAA+O), thioacetamide + C60 fullerene lower dose (TAA+O+F1), and thioacetamide + C60 fullerene higher dose (TAA+O+F2).

HMGB1, whole liver homogenate, X-ray film

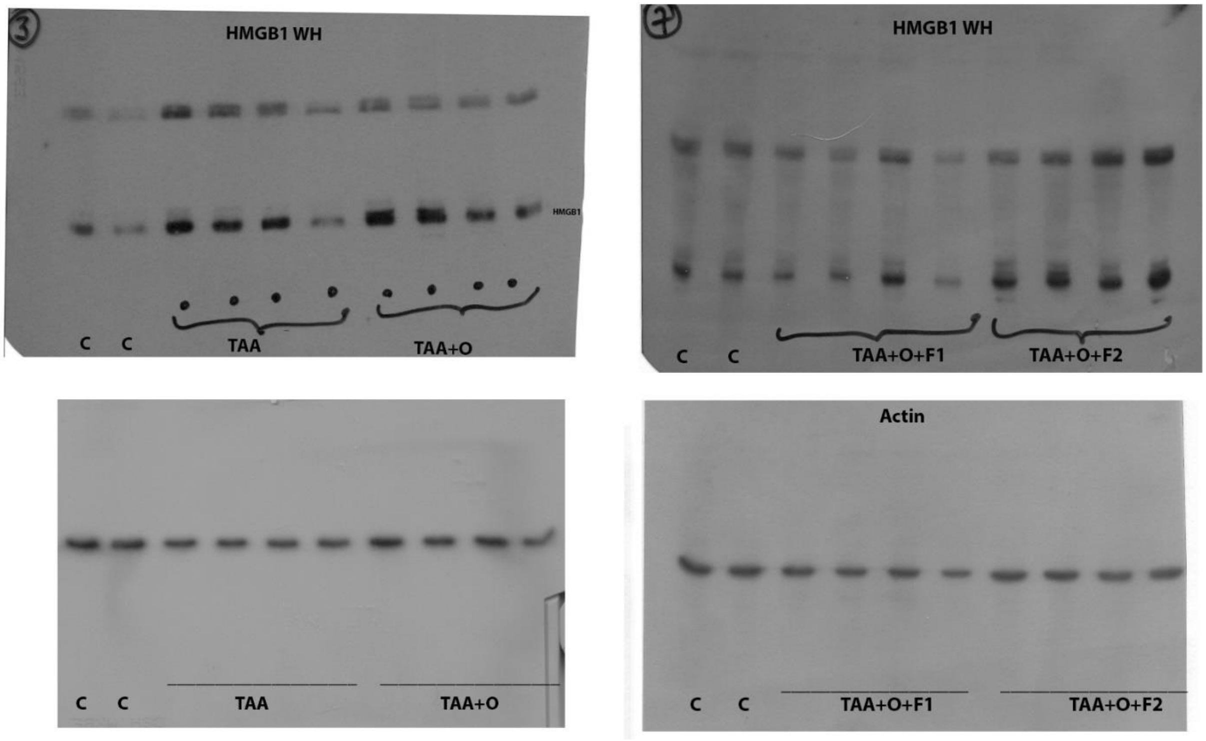

2. Original images of Western blot used in Figure 4. Protein expression levels of phospo-NF-kB p65 in whole liver homogenates in control, and rats treated with thioacetamide (TAA), thioacetamide + virgin olive oil (TAA+O), thioacetamide + C60 fullerene lower dose (TAA+O+F1), and thioacetamide + C60 fullerene higher dose (TAA+O+F2).

Phospho-NFkB p65, whole liver homogenate, IBright detection

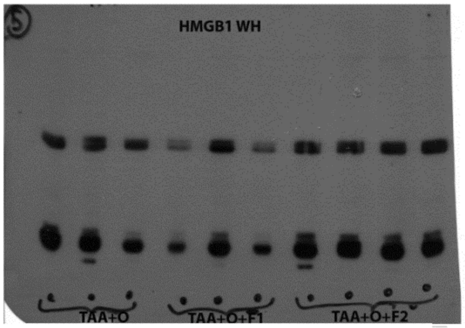

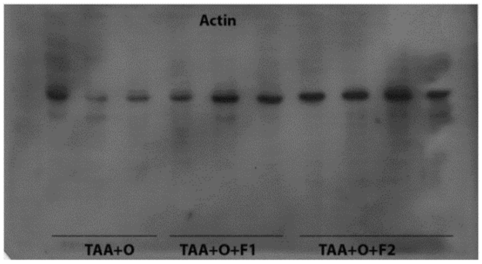

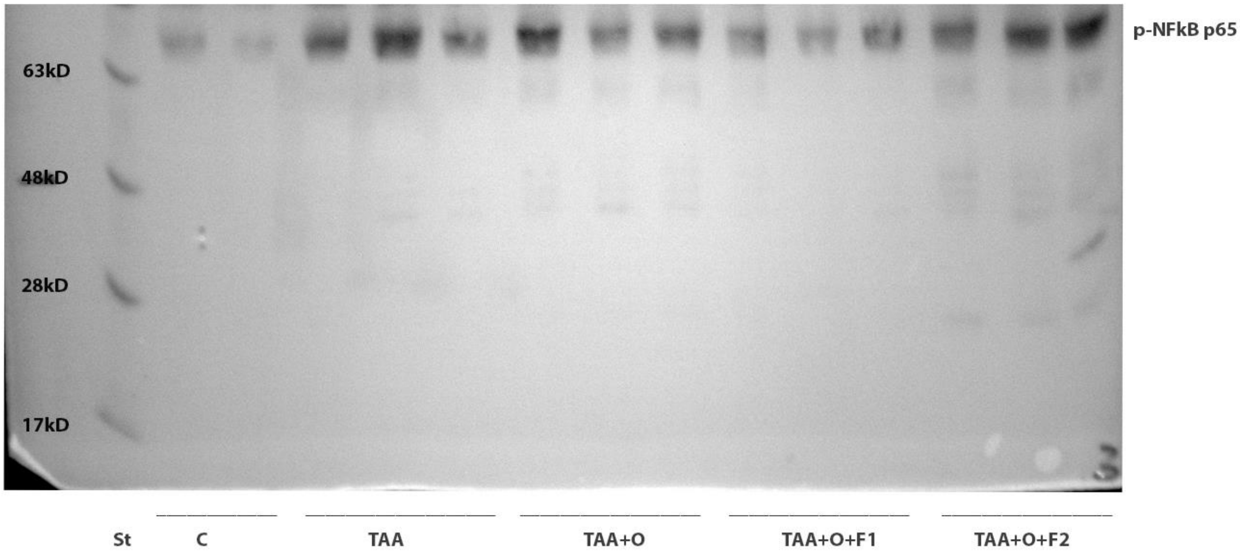

3. Original images of Western blot used in Figure 5A. Protein expression levels of haeme oxygenase-1 (HO-1) in whole liver homogenates in control, and rats treated with thioacetamide (TAA), thioacetamide + virgin olive oil (TAA+O), thioacetamide + C60 fullerene lower dose (TAA+O+F1), and thioacetamide + C60 fullerene higher dose (TAA+O+F2).

HO-1, whole liver homogenate, IBright detection

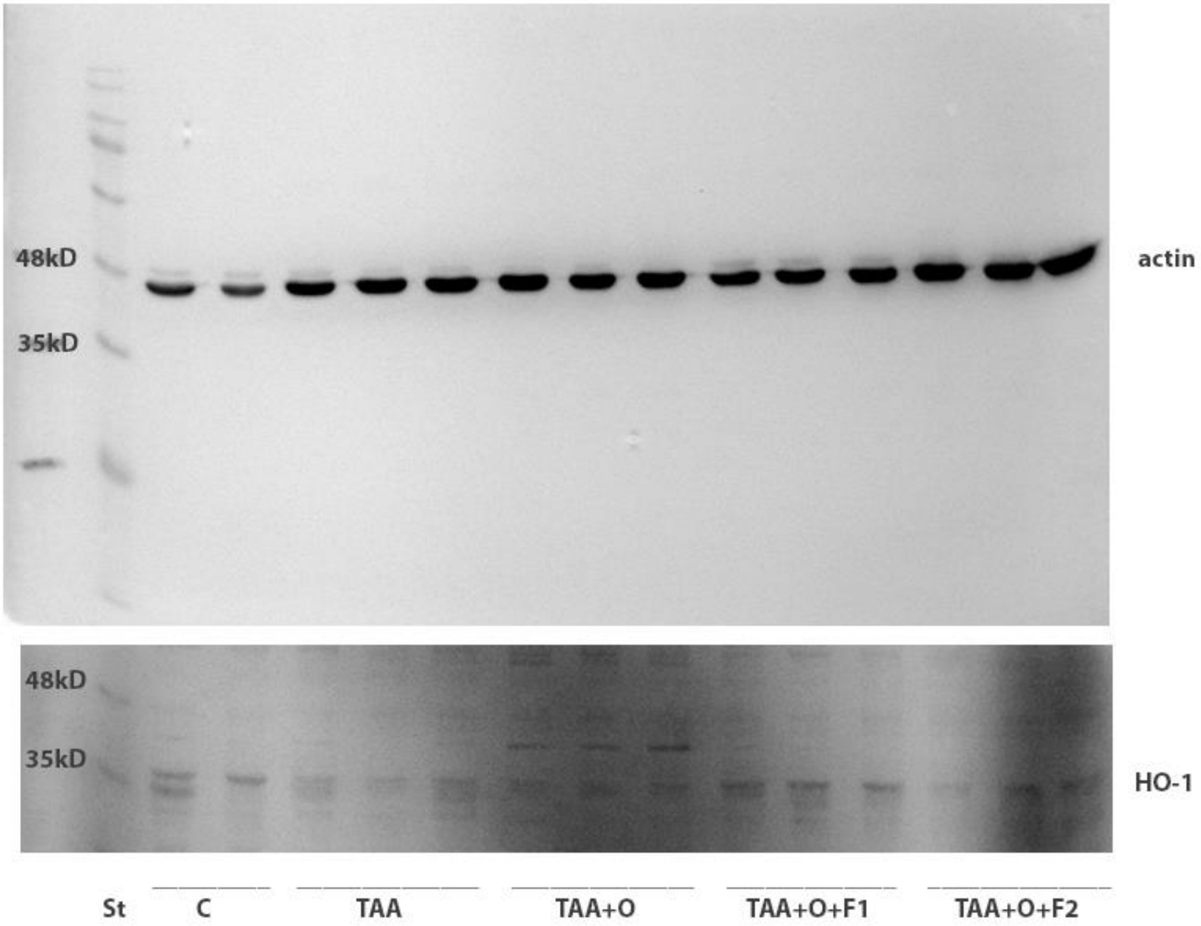

4. Original images of Western blot used in Figure 5A. Protein expression levels of manganese superoxide-dismutase (MnSOD) and copper-zinc superoxide-dismutase (CuZnSOD) in whole liver homogenates in control, and rats treated with thioacetamide (TAA), thioacetamide + virgin olive oil (TAA+O), thioacetamide + C60 fullerene lower dose (TAA+O+F1), and thioacetamide + C60 fullerene higher dose (TAA+O+F2).

MnSOD, whole liver homogenate, IBright detection

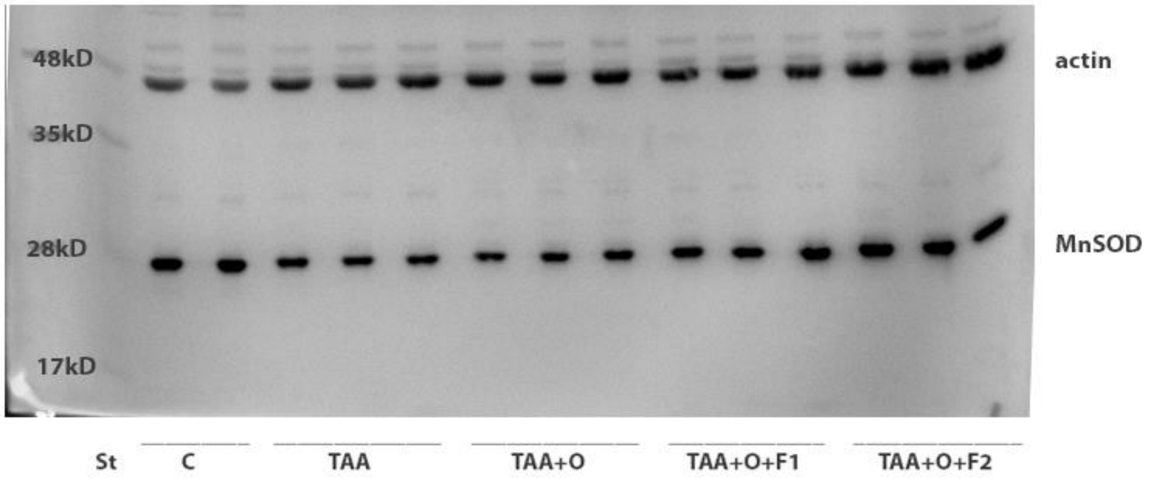

CuZnSOD, whole liver homogenate, IBright detection

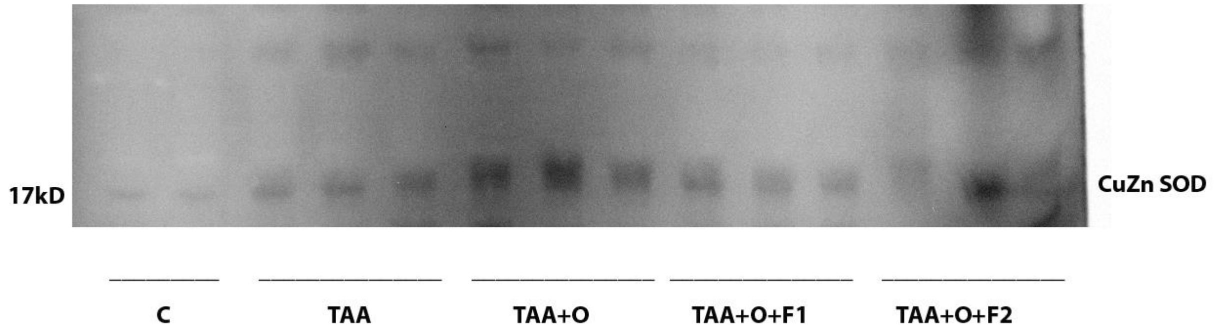

5. Original images of Western blot used in Figure 5B. Protein expression levels of total Nrf2 and NF-kB p65 in nuclear extracts in control, and rats treated with thioacetamide (TAA), thioacetamide + virgin olive oil (TAA+O), thioacetamide + C60 fullerene lower dose (TAA+O+F1), and thioacetamide + C60 fullerene higher dose (TAA+O+F2).

Total NRF-2 and NF-kB p65 ratio, nuclear liver extracts, IBright detection

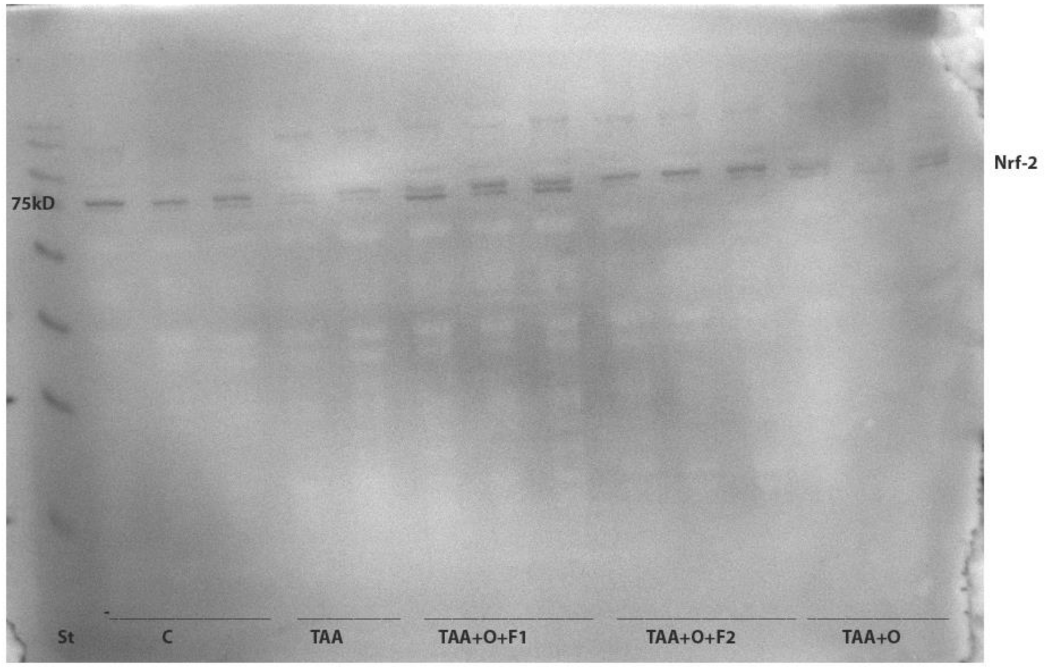

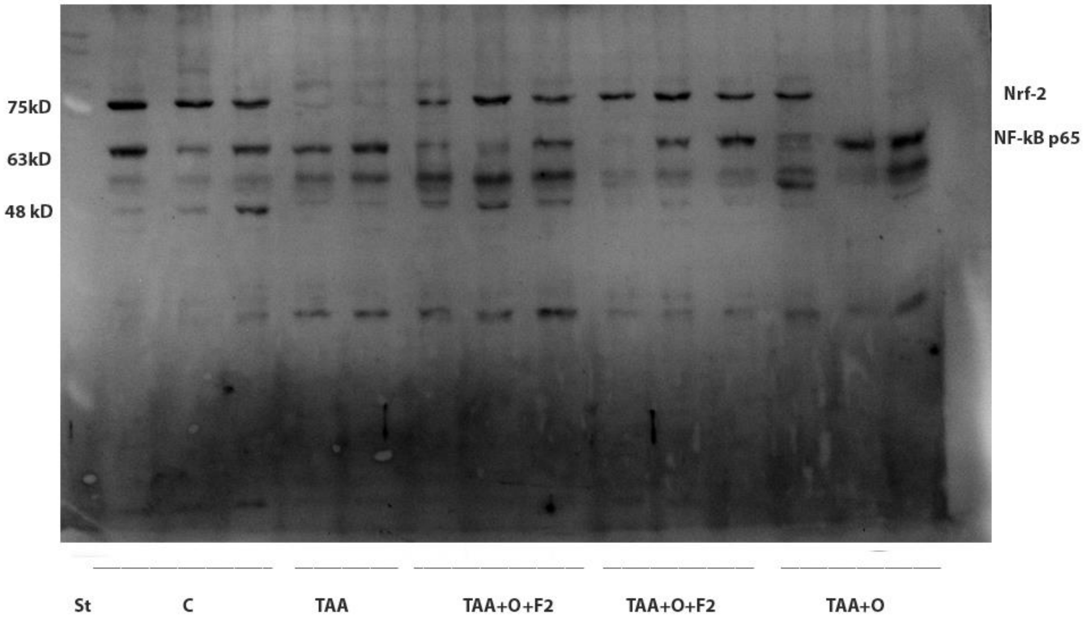

Supplement: Supplementary file 1 [file antioxidants-10-00911-s001.zip › antioxidants-1204538-supplementary.pdf]
